# Pharmacogenetics Meets Metabolomics: Discovery of Tryptophan as a New Endogenous OCT2 Substrate Related to Metformin Disposition

**DOI:** 10.1371/journal.pone.0036637

**Published:** 2012-05-08

**Authors:** Im-Sook Song, Do Yup Lee, Min-Hye Shin, Hyunmi Kim, Yun Gyong Ahn, Inmyoung Park, Kyoung Heon Kim, Tobias Kind, Jae-Gook Shin, Oliver Fiehn, Kwang-Hyeon Liu

**Affiliations:** 1 Department of Pharmacology and PharmacoGenomics Research Center, Inje University College of Medicine, Busan, Korea; 2 Genome Center, University of California Davis, Davis, California, United States of America; 3 School of Life Sciences and Biotechnology, Korea University, Seoul, Korea; 4 Seoul Center, Korea Basic Science Institute, Seoul, Korea; 5 Department of Land, Air and Water Resources, University of California Davis, Davis, California, United States of America; 6 College of Pharmacy and Research Institute of Pharmaceutical Sciences, Kyungpook National University, Daegu, Korea; University of Ulster, United Kingdom

## Abstract

Genetic polymorphisms of the organic cation transporter 2 (OCT2), encoded by *SLC22A2*, have been investigated in association with metformin disposition. A functional decrease in transport function has been shown to be associated with the OCT2 variants. Using metabolomics, our study aims at a comprehensive monitoring of primary metabolite changes in order to understand biochemical alteration associated with OCT2 polymorphisms and discovery of potential endogenous metabolites related to the genetic variation of OCT2. Using GC-TOF MS based metabolite profiling, clear clustering of samples was observed in Partial Least Square Discriminant Analysis, showing that metabolic profiles were linked to the genetic variants of OCT2. Tryptophan and uridine presented the most significant alteration in *SLC22A2-808TT* homozygous and the *SLC22A2-808G>T* heterozygous variants relative to the reference. Particularly tryptophan showed gene-dose effects of transporter activity according to OCT2 genotypes and the greatest linear association with the pharmacokinetic parameters (Cl_renal_, Cl_sec_, Cl/F/kg, and Vd/F/kg) of metformin. An inhibition assay demonstrated the inhibitory effect of tryptophan on the uptake of 1-methyl-4-phenyl pyrinidium in a concentration dependent manner and subsequent uptake experiment revealed differential tryptophan-uptake rate in the oocytes expressing OCT2 reference and variant (808G>T). Our results collectively indicate tryptophan can serve as one of the endogenous substrate for the OCT2 as well as a biomarker candidate indicating the variability of the transport activity of OCT2.

## Introduction

The rapidly growing field of metabolomics has been regarded as the most efficient and direct approach for validating biochemical functions of putative enzymes and transporters, identifying ligands for unknown enzyme functions, and identifying and quantifying endogenous and exogenous chemicals in tissues and body fluids [1]–[5]. This technology has been applied to explore the protein activity and substrate selectivity of serine hydrolase activities in complex proteomes [6] and to identify important lipid substrates of fatty acid amide hydrolase using solution-based *in vitro* enzyme assays [7]. The technology has been further extended to genome-wide association studies of metabolic phenotypes related to quantitative variations of single-nucleotide polymorphisms and endogenous organic compounds in human serum [8].

Organic cation transporter 2 (OCT2), encoded by *SLC22A2*, is important in the elimination of organic cations. The organic cation substrates of OCT2 include model organic cations such as tetraethylammonium (TEA) and decynium 22; clinically important therapeutic drugs such as metformin [9], procainamide, cisplatin, citalopram, and cimetidine; endogenous compounds such as dopamine and norepinephrine; and toxic metabolites such as pyridinium metabolites of haloperidol and 1-methyl-4-phenyl pyridinium (MPP^+^) [10]–[12]. As OCT2 is expressed primarily in the basolateral membrane of kidney proximal tubules, it is expected to play a leading role in renal elimination of exogenous and endogenous substrates [10]–[12]. [13]–[15].

The *SLC22A2-808G>T* variant has been identified with an allele frequency higher than 10% in all ethnic groups [12]–[14]. It has been investigated as a contributing factor to the inter-individual variation in the disposition and distribution of substrate drugs [13]–[15]. The intrinsic clearances of MPP^+^ and metformin, substrates for OCT2, were decreased in oocytes, MDCK and HEK-293 cells overexpressing the *SLC22A2-808G>T* variant protein compared with the reference protein, indicating that this genetic variation decreased the transport activity of OCT2 [13]–[16]. As expected from the primary distribution of OCT2 in the kidney, the *808G>T* variant affected mainly tubular excretion and renal clearance, increasing the plasma metformin level in subjects expressing the *808G>T* variant. The peak plasma concentration (C_max_) of metformin was increased 1.62-fold in the *SLC22A2-808TT* homozygous variant, and the area under the plasma concentration versus time curve (AUC_last_) was increased 1.46- and 1.74-fold in *SLC22A2-808GT* heterozygous and 808TT homozygous groups, respectively. The active secretion clearance (Cl_sec_) of metformin is believed to represent an OCT2-mediated process and is obtained by subtracting the creatinine clearance (Cl_cr_) from the renal clearance (Cl_renal_) of metformin. The Cl_sec_ of metformin was decreased by 37.7% and 61.8% in the *SLC22A2-808GT* and *808TT* variants, respectively, compared with the reference genotype group [17].

To monitor the biochemical traits of the genetic OCT2 variants and identify potential endogenous substrates, we performed a non-targeted assignment of substrates to the transporter using gas chromatography-mass spectrometry (GC-MS)-based metabolite profiling. First, by comparing the levels of endogenous urine metabolites among individuals with different OCT2 genotypes, we showed that individuals expressing different OCT2 variants had significantly dissimilar metabolic capacities. Then, all of the identified metabolites and unknown compounds exhibiting significant differences among the OCT2 variants were subjected to regularized canonical correlation analysis (rCCA) to identify correlations with various pharmacokinetic (PK) parameters of metformin. Finally, *in vitro* functional studies of inhibitory effects and uptake rates demonstrated that tryptophan can be an endogenous substrate of OCT2.

## Results

### 1. Metabolite profiles associated with different OCT2 variants indicate putative biomarkers for the variability of OCT2 transport activity

#### OCT2 variants induce differential metabolome composition and phenotype

We performed metabolite profiling of urine samples obtained from carriers of three different *SLC22A2* genotypes. The genotypes of the 21 subjects were *SLC22A2* reference (7 males, 2 females), 808GT heterozygous (4 males, 2 females), and 808TT homozygous (4 males, 2 females) (Spreadsheet S1). A total of 85 metabolic signals were structurally identified by GC-MS using the BinBase algorithm, with 80% occurrence in at least one class of samples, which automatically excluded known artifacts such as phthalates or polysiloxanes. The endogenous metabolites included amino acids, carbohydrates, amines, fatty acids, and organic acids. Particularly, it includes xanthine, xanthosine, and uridine that are at very low cellular levels in urine, whose detection was advantaged by high sensitivity and scan rate of time-of-flight mass spectrometry technology along with advanced deconvolution algorithm but it is not likely to be the case for other analytical platform.

**Figure 1:**
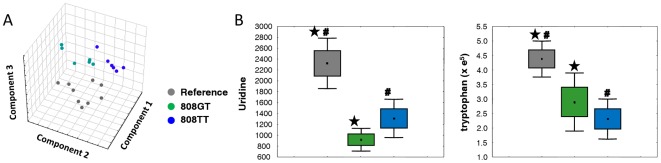
Statistical analysis of urine metabolome of three different *SLC22A2* genotypes. (A) Supervised multivariate analysis of urine metabolite profiles among *SLC22A2* genotypes. Partial least square analysis was performed with metabolomic profiles of urine samples. Component 1 indicates principal component 1 that can explain the most variation in dataset. Likewise, component 2 and component 3 indicate principal component 2 and 3 respectively. Grey, green, and blue colors present the *SLC22A2* reference, heterozygous, and homozygous genotype groups respectively. (B) Multi-comparison analysis of uridine and tryptophan in urine from individuals with three *SLC22A2* genotypes. Of the metabolites showing significant differences among genotypes, uridine (left panel) and tryptophan (right panel) were the most significantly decreased in *SLC22A2 808GT* and *808TT*, respectively. Data, assuming normal distributions, are displayed by box-whisker plots, giving the arithmetic mean for each category, the standard error as a box, and whiskers for 1.96 times the category standard error to indicate the 95% confidence intervals. Bars sharing a symbol are different at *p*<0.05 by Scheffé's post-hoc test.

Multivariate analysis showed that the principal factors differentiating the metabolic profiles were associated with the genetic variants of *SLC22A2*. Only supervised partial least squares (PLS) analysis could separate the three different genotypes with application of the first three components (Figure 1A). Unsupervised principal components analysis (PCA) only obscurely differentiated the metabolite phenotypes between the reference group and both variants (Figure S1). Univariate analysis of the variance was used to interrogate compositional changes in metabolites associated with *SLC22A2* variants. Six metabolites were significantly changed (*p*<0.01, Student's *t*-test, Spreadsheet S1) in homozygotes compared with the reference group. Among those, tryptophan showed the most significant alteration (*p* = 0.00086). Other altered metabolites were oxoproline, 3-hydroxy-3-methylglutaric acid, glucose, 2-hydroxyvaleric acid, and uridine. In a comparison between heterozygotes and the reference group, uridine was altered most drastically (*p* = 0.0005); 13 other endogenous metabolites (*p*<0.01; Student's *t*-test; Spreadsheet S1) and tryptophan (*p* = 0.0028) also showed differences. Multi-comparison analysis (Scheffé's test) supported a significant alteration in both tryptophan and uridine, with the highest levels of downregulation associated with each OCT2 variant compared with the reference group (Figure 1B, C). These metabolites were endogenous substrate candidates potentially related to the transport activity of OCT2.

#### Canonical correlation analysis reveals a strong association between endogenous metabolites and PK parameters of metformin

In the next approach, we applied canonical correlation statistics to analyze the interactions between metabolite compositions and PK parameters of metformin, which was previously published by our laboratory [17]. Canonical correlation analysis (CCA) is a multivariate analysis often applied in psychological, climate, and ecological studies to quantify the correlations between two separate data sets measured on the same experimental units [18], [19]. In the analysis, 85 identified metabolites were introduced to regularized CCA with the following eight metformin PK parameters: C_max_, AUC_last_, elimination rate constant (K_e_), body weight-normalized clearance (Cl/F/kg), body weight-normalized volume of distribution (Vd/F/kg), renal clearance (Cl_renal_), creatinine clearance (Cl_cre_), and secretion clearance (Cl_sec_). The complete list of the metabolites and metformin PK parameters is provided in Spreadsheet S1.

**Figure 2:**
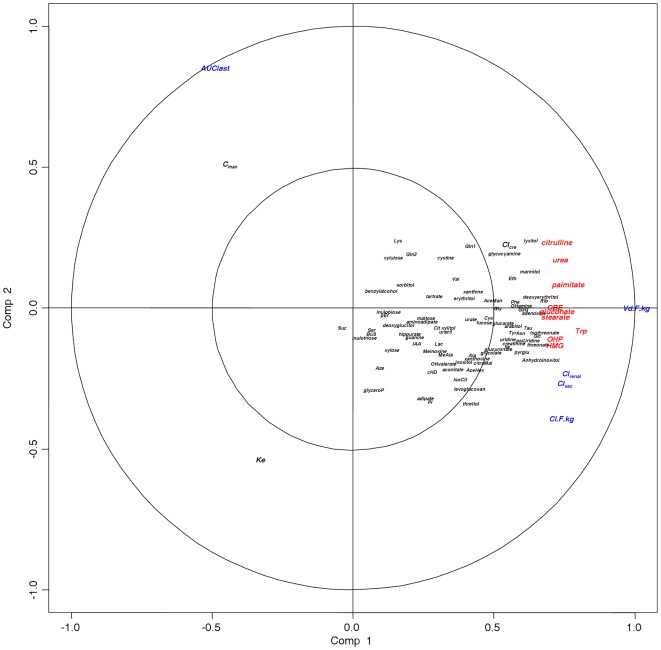
Visualization of the rCCA results of metabolites and pharmacokinetic parameters involved in metformin disposition. The canonical structure correlations of 85 metabolites detected by GC-MS, and eight PK parameters with the first two canonical variates show a distinct association between metabolites with PK parameters. The metabolites and PK parameters that presented higher than the threshold (canonical correlation, >0.7) are colored in red and blue, respectively. The metabolites are tryptophan, citrulline, urea, free fatty acids (palmitate and stearate), 3-hydroxy-3-methylglutaric acid, 3-hydroxypropionic acid, and conduritol-beta-epoxide. The PK parameters positively associated with the metabolite cluster are Vd/F/kg, Cl_renal_, Cl_sec_, and Cl/F/kg. Both AUC_last_ and C_max_ were located in the opposite direction from the cluster. HMG, 3-hydroxy-3-methylglutaric acid; Trp, tryptophan; OHP, 3-hydroxypropionic acid; CBE, conduritol-beta-epoxide; C_max_, peak plasma concentration; AUC_last_, area under the plasma concentration vs. time curve; K_e_, elimination rate constant; Cl/F/kg, body weight-normalized clearance; Vd/F/kg, body weight-normalized volume of distribution; Cl_renal_, renal clearance; Cl_cre_, creatinine clearance; Cl_sec_, secretion clearance.

Figure 2 shows the CCA variables plot applied to the combined data set, including all three *SLC22A2* genotypes. The graphical result of the canonical correlation with the first two canonical dimensions (see Materials and Methods section) shows spatial closeness between metabolites and metformin PK parameters. Some variables were distinguished where the components (metabolites and metformin PK parameters) were projected in the same direction from the origin. The variables were nine metabolites and four metformin PK parameters, including Cl_renal_, Cl_sec_, Cl/F/kg, and Vd/F/kg (canonical correlation, >0.70). Among the clustered metabolites, tryptophan showed the greatest distance from the origin, with close spatial relatedness to the metformin PK parameters (Cl_renal_, Cl_sec_, Cl/F/kg, and Vd/F/kg); among the metformin PK parameters, Vd/F/kg showed the greatest distance from the origin, implying the strongest correlation with the metabolites. Three metabolites of the clustered group, tryptophan, citrulline, and urea, were amine group-containing compounds. The others were free fatty acids (palmitate and stearate), 3-hydroxy-3-methylglutaric acid (HMG), and 3-hydroxypropionic acid. No solid associations were observed for the PK parameters and remaining metabolites including uridine, which presented the greatest alteration in OCT2 variant, nor pseudouridine, which shares structural similarity with uridine but exists at the highest abundance among urine metabolites. Instead, AUC_last_ and C_max_ were located in the opposite direction from the main cluster, indicating a moderate level of negative correlation with some of the metabolites.

**Figure 3:**
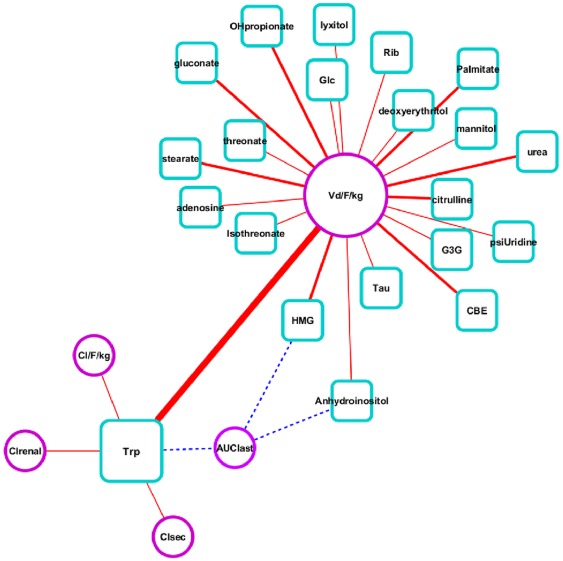
Network visualization of rCCA results. The network was constructed using a pairwise linkage score between metabolites and metformin PK parameters. Red lines represent positive correlations (canonical correlation, >0.65), and the thickness is proportional to the level of linearity. Nodes for metabolites and PK parameters are squares (blue) and circles (purple), respectively, and the size of node indicates the level of connectivity. Blue dot lines were additionally linked in order to present negative association which is below the threshold. Trp, tryptophan; Rib, ribose; Glc, glucose; Tau, taurine; HMG, 3-hydroxy-3-methylglutaric acid; G3G, glycerol-3-galactoside; Anhydroinositol, 1,2-anhydro-myo-inositol; deoxyerythritol, 2-deoxyerythritol; OHP, 3-hydroxypropionic acid; psiUridine, pseudo-uridine.

To analyze the pairwise associations between metabolites and metformin PK parameters in more detail, we constructed a network based on the correlation results. In the network, the variables X–Y (metabolites-PK parameters) represent relevant associations, and the network calculates the similarity between X and Y in a pair-wise manner. As observed in Figure 3, Vd/F/kg formed the highest degree of connectivity with all 29 endogenous metabolites (canonical correlation, >0.60), and the strongest association was with tryptophan (canonical correlation, 0.804). Among the metabolites, tryptophan had the highest degree of positive correlation with Cl_renal_, Cl_sec_, and Cl/F/kg.

Additionally, we considered unknown compounds for linear associations with the PK parameters of metformin. Forty-three unknown compounds with the most significant differences among the *SLC22A2* genotypes (*p*<0.001, Spreadsheet S1) were added to the metabolite list for the CCA analysis. As a result, four compounds were significantly correlated with the PK parameters (canonical correlation, >0.70). Their ID numbers were 234622, 267701, 267652, and 224529 (http://eros.fiehnlab.ucdavis.edu:8080/binbase-compound/database/select Database). Nevertheless, among all metabolite variables considered, tryptophan still presented the greatest association with Vd/F/kg and the highest connectivity with the other PK parameters (Figure S2).

### 2. *In vitro* functional studies suggest that tryptophan can serve as an endogenous substrate for OCT2

#### Tryptophan inhibition of OCT2-mediated MPP^+^ uptake

Based on the metabolite profiling outcome, we used some of the metabolites (glycine, taurine, uridine, and tryptophan) in further *in vitro* functional studies. Uridine and tryptophan were selected because their levels were the most significantly downregulated in each OCT2 variant group compared with the reference group. In particular, the downregulation of tryptophan was intensified according to the genotype. Tryptophan was also highly correlated with four metformin PK parameters (Cl_renal_, Cl_sec_, Cl/F/kg, and Vd/F/kg). Glycine and taurine, which were significantly altered, but showed low and moderate levels of correlation with metformin PK parameters respectively, were used as negative controls for OCT2 uptake.

**Figure 4:**
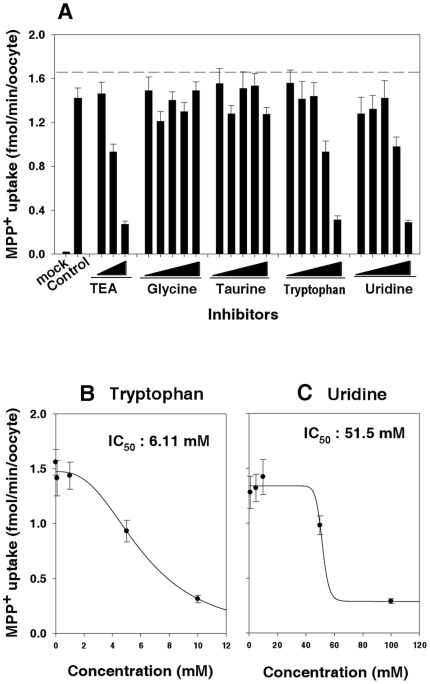
Comparison of the inhibition of OCT2-mediated MPP^+^ uptake by the metabolites at multiple concentration ranges. (A) Inhibitory effect of TEA (0.1–1000 mM), glycine (1–100 mM), taurine (1–100 mM), L-tryptophan (0.01–10 mM), and uridine (1–100 mM) on OCT2-mediated MPP^+^ uptake. (B) Concentration-dependent inhibitory effect of L-tryptophan (0.01–10 mM) on the uptake of 18.5 nM [^3^H]MPP^+^ in oocytes expressing OCT2. (C) Concentration-dependent inhibitory effect of uridine (1–100 mM) on the uptake of 18.5 nM [^3^H]MPP^+^ in oocytes expressing OCT2. IC_50_ was calculated by non-linear regression analysis using an inhibitory effect E_max_ model: v = E_max_−E_max_×C/(IC_50_+C). Data represent means ± standard error (SE) of eight independent experiments.

The ability of OCT2 to bind the different metabolites was quantified by measuring the inhibitory effect of glycine (1–100 mM), taurine (1–100 mM), uridine (1–100 mM), and tryptophan (0.01–10 mM) on OCT2-mediated uptake of MPP^+^ in oocytes expressing OCT2. TEA (0.01–1 mM), a known OCT2 inhibitor, was included as a positive control. Figure 4 compares the inhibition of OCT2-mediated uptake by the metabolites at multiple concentrations. Glycine and taurine at concentrations of up to 100 mM showed no inhibition of MPP^+^ uptake. Tryptophan showed the strongest inhibition of MPP^+^ uptake, with an IC_50_ of 6.11 mM. Uridine had a weaker inhibitory effect, with an IC_50_ of 51.5 mM, which is 8.43-fold that of tryptophan. This result motivated further examination of tryptophan.

#### Differential tryptophan uptake in oocytes overexpressing the OCT2 reference and 808G>T variant

The inhibition of OCT2 substrate uptake by tryptophan suggested a strong affinity of OCT2 for tryptophan. Therefore, we investigated the differential rate of tryptophan uptake by the OCT2 reference and 808G>T variant overexpressed in oocytes. Eight independent replicates were analyzed, and each experiment included uptake measurements of MPP^+^ as a representative OCT2 substrate. The uptakes of tryptophan and MPP^+^ mediated by the OCT2 reference and variant were calculated by subtracting the uptake rate in water-injected oocytes from that in OCT2-expressing oocytes.

**Figure 5:**
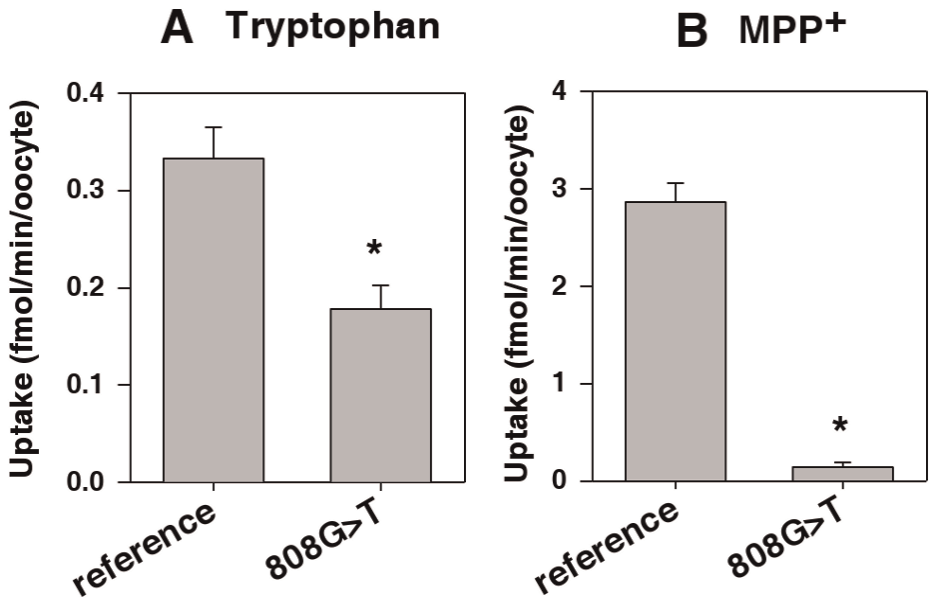
Differential tryptophan uptake rate in oocytes overexpressing the OCT2 reference and 808G>T variant. Uptake of (A) 100 nM [^3^H]tryptophan or (B) 18.5 nM [^3^H]MPP^+^ by oocytes overexpressing the OCT2 reference or 808G>T variant. Defolliculated oocytes were injected with 50 ng of capped cRNA and incubated for 2 days. Uptake was measured for 30 min. Data represent means ± SE of eight independent experiments. * *P*<0.05, compared with OCT2 reference using Student's *t*-test.

The uptake of both MPP^+^ and tryptophan increased linearly for 1 h, and uptake measurements at 30 min were used for the analysis. As expected, MPP^+^ uptake was lower for the 808G>T variant than for the reference protein (Figure 5). Within 30 min, the rate was dramatically reduced from 2.9 to nearly 0 fmol/min/oocyte overexpressing the 808G>T variant, consistent with what we found in a previous study [15]. Tryptophan uptake also displayed a lower translocation rate for the 808G>T variant, with a significant reduction from 0.3 to 0.14 fmol/min/oocyte (2.0-fold; *p*<0.05).

### 3. Substrate specificity of OCT2 for tryptophan compared with that of other transporters

**Figure 6:**
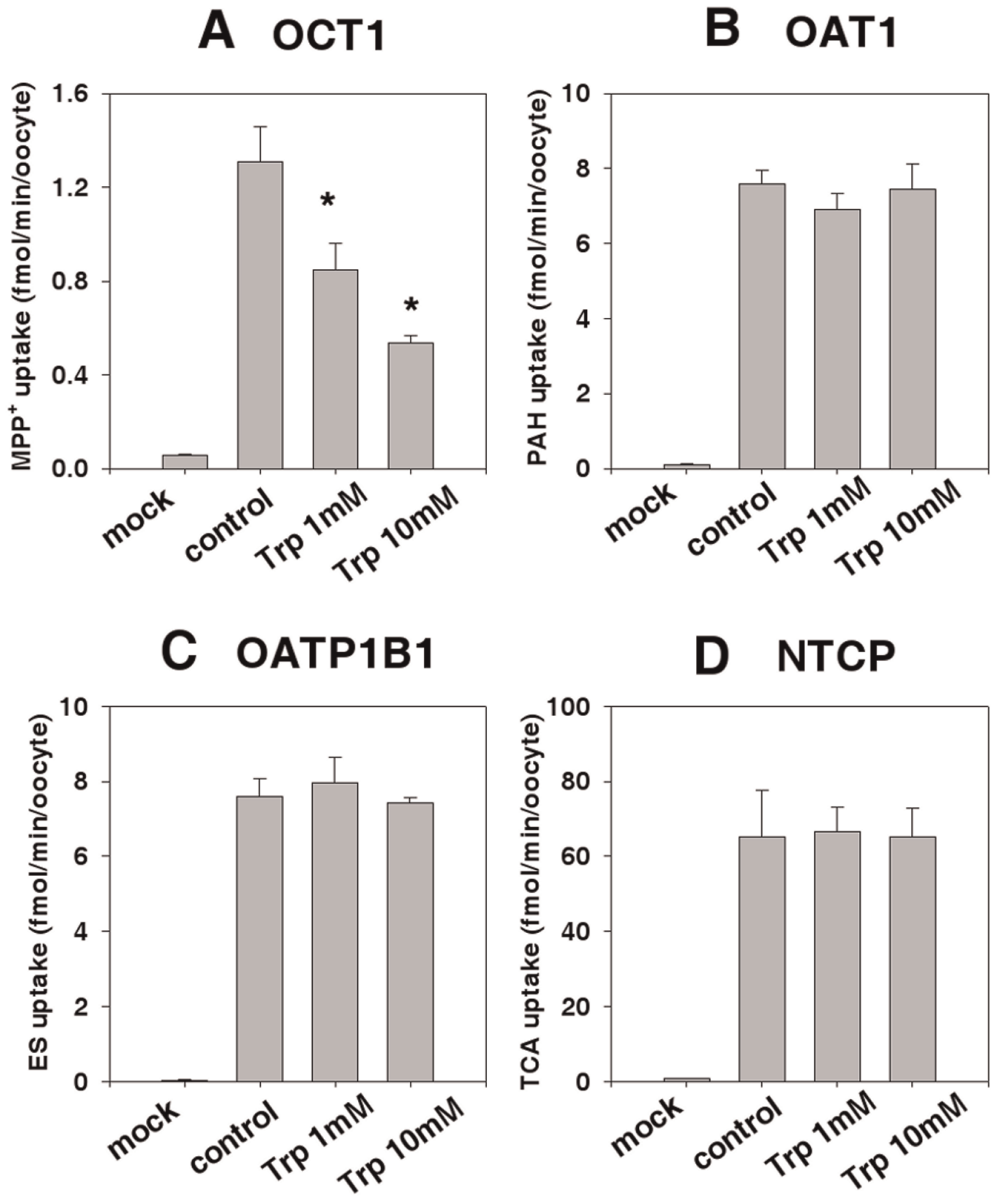
Inhibitory effect of tryptophan on transporter uptake. Inhibitory effect of 1 or 10 mM tryptophan on (A) OCT1-mediated MPP^+^ uptake (18.5 nM), (B) OAT1-mediated PAH uptake (400 nM), (C) OATP1B1-mediated ES uptake (18.5 nM), and NTCP-mediated TCA uptake (1.5 µM). Mock: Water-injected oocyte. Control: Oocytes expressing OCT1, OAT1, OATP1B1, or NTCP without tryptophan. Trp: Tryptophan. Data represent means ± SE of eight independent experiments. * *P*<0.05, compared with water-injected oocytes using Student's *t*-test.

The substrate specificity of OCT2 for tryptophan was compared with that of other transporters by testing tryptophan as an inhibitor of four other transporters, OCT1, organic anion transporter 1 (OAT1), organic anion transporting polypeptide 1B1 (OATP1B1), and Na^+^/taurocholate co-transporting polypeptide (NTCP), using MPP^+^, para-aminohippurate (PAH), estrone-3-sulfate (ES), and taurocholic acid (TCA), respectively, as the respective typical substrates. For the inhibition assay, the uptake of each substrate in oocytes overexpressing the appropriate transporter was measured in the presence and absence of tryptophan at 0, 1.0, and 10.0 mM. Tryptophan significantly inhibited MPP^+^ uptake by OCT1, but it did not alter OAT1-mediated PAH uptake (400 nM), OATP1B1-mediated ES uptake (18.5 nM), or NTCP-mediated TCA uptake (1.5 µM) (Figure 6). The significant inhibitory effect of tryptophan on OCT1-mediated uptake of MPP^+^ occurred in a concentration-dependent manner, with 50% and 70% inhibition at 1 and 10 mM tryptophan, respectively.

Tryptophan translocation rates were also measured using the same transporters as those used in the inhibition assay. OCT1-facilitated translocation of tryptophan was observed, with a rate of 0.85 fmol/min/oocyte (Figure S3).

### 4. Absolute quantification of tryptophan in urine samples, by liquid chromatography-coupled tandem mass spectrometry

**Figure 7:**
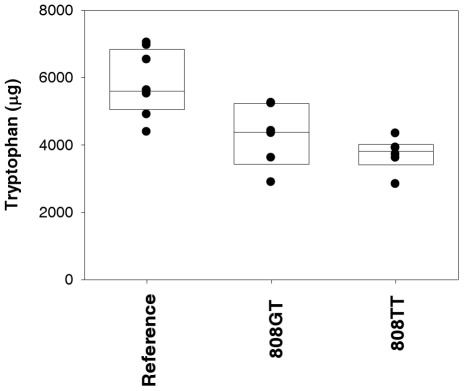
Absolute levels of tryptophan in urine samples. Tryptophan levels were measured from *SLC22A2* reference (*n* = 9), *808GT* (*n* = 6), and *808TT* (*n* = 6) carriers using selected reaction monitoring with liquid chromatography-coupled tandem mass spectrometry. The boundary of the box closest to zero indicates the 25^th^ percentile, a line within the box marks the median, and the boundary of the box farthest from zero indicates the 75^th^ percentile.

Tryptophan levels in the urine samples of individuals carrying different *SLC22A2* genotypes were absolutely quantified by selected reaction monitoring with liquid chromatography-coupled tandem mass spectrometry (LC-MS/MS). As shown in Figure 7, the absolute amounts of tryptophan in urine samples differed significantly according to *SLC22A2* genotype, with the highest level measured in the reference group (5786±968 µg), followed in order by the *808GT* (4287±920 µg) and *808TT* groups (3718±499 µg).

## Discussion

The goal of the present study was to identify endogenous substrates of the transporter OCT2 by 1) detecting metabolites differentially present in urine samples collected from carriers of three different *SLC22A2* genotypes (reference group, heterozygotes, and homozygotes); 2) determining linear associations between the altered metabolites and metformin PK parameters; and 3) evaluating the inhibitory potential and substrate specificity of the identified metabolites *in vitro*.

Altered metabolite levels in urine samples may reflect differential activity of transporters. To detect differentially regulated metabolites among three *SLC22A2* genotypes, we applied non-targeted metabolite profiling in urine samples from carriers of the reference *SLC22A2* genotype and two variant genotypes, *808GT* and *808TT*. Uridine and tryptophan were the most significantly decreased compounds associated with the *808GT* and *808TT* variants, compared with the levels in the reference group. Tryptophan showed a gradual decrease according to the order of genetic variation (reference group>*808GT* heterozygotes>*808TT* homozygotes), suggesting a gene-dose effect on OCT2 transporter activity, which may be specific for tryptophan.

The disposition of metformin has been reported to be affected by *SLC22A2* genotype, leading us to hypothesize that metabolites closely correlated with metformin PK parameters may be endogenous substrates of OCT2. Linear associations between the metabolite levels and metformin PK parameters were determined by CCA, which allows for quantifying the level of coordination between two different parametric readouts. Among the group of metabolites found to be closely associated with four metformin PK parameters (Cl_renal_, Cl_sec_, Cl/F/kg, and Vd/F/kg; canonical correlation, >0.70), tryptophan showed the greatest linear relatedness to all four, especially to Vd/F/kg (canonical correlation, 0.804). Tryptophan also demonstrated a moderate negative correlation with AUC_last_. The strong correlations between the tryptophan level and metformin PK parameters indicated that it may be a potential substrate of OCT2.

Prior to *in vitro* function study, which is the comparably slow and laborious stage, we validated the significance of the findings by comparing the result with other type of data normalization, creatinine-level. Recent publications report the limited application of creatinine level-normalization due to the significant variability of the levels depending on age, gender, and time point [20]–[22]. However, we selected the method since it has been a common method along with urine-volume normalization [23], [24], and also different statistics can provide more reliable data interpretation [25]. In general, we reproduced the compatible pattern to the urine volume-normalized result such as compatibility of normalization factor and variable distribution (Text S1). At the same time, we observed the dissimilarity between datasets differentially normalized such as the detailed list of significantly altered compounds, and also the superiority of reproducibility in biological replicate in the urine volume-normalized data set (Text S1). Nonetheless, we selected tryptophan for following in vitro function study based on its greatest alteration with gene-dose effect (in urine normalization) and the strongest linkage to the PK parameters (in both normalization). Uridine and taurine were also considered since they showed consistent result in univariate analysis (the greatest significance) and rCCA in which moderate levels were found in the association with the PK parameters. Note that the consistency of rCCA outcome implies a great advantage of the multivariate statistics (rCCA) over univariate statistics that could bias result interpretation driven by data normalization types. We further narrowed down the candidates based on prior knowledge and simultaneous consideration of two statistical criteria (significant alteration and strong association with the PK parameters of metformin). For example, we did not consider xanthine and xanthosine because they both indicated very poor association with the PK parameters. Citrulline was not included for the function study since it has been reported as the substrate for organic anion transporter 1 (OAT1) [26]. Instead, glycine was further assessed as a representative negative control as it only presented the significant alteration but weak correlation with the PK parameters so that we can evaluate the benefit of additional statistics, regularized-canonical correlation analysis (rCCA).

Accordingly, in the evaluation of their ability to inhibit MPP+ uptake by OCT2, tryptophan showed the greatest inhibition, with an IC_50_ of 6.11 mM while merely moderate inhibitory effect was detected in uridine with a much higher IC_50_ (51.5 mM) and no effect in taurine and glycine. This in vitro function result circuitously evidenced the suitability of data analysis preferentially used in our study (urine volume-wise normalization), and the powerful benefit of rCCA statistics which can precisely predict the potential candidate of a transporter by characterizing linkage of metabolites and the pharmacokinetic parameters associated with metformin disposition. The following tryptophan uptake experiments in oocytes overexpressing the OCT2 reference and 808G>T variant confirmatively revealed significantly lower uptake and a significantly slower translocation rate with the variant protein than with the reference protein.

In subsequent inhibition assay to verify the specificity of tryptophan for OCT2, tryptophan inhibited OCT1-mediated uptake of MPP^+^, but not the transport function of OAT1, OAT1B1, or NTCP. In tryptophan uptake experiments, OCT1 facilitated the translocation of tryptophan, whereas OAT1, OAT1B1, and NTCP did not. OCT1 and OCT2 share about 70% sequence similarity and have a similar broad range of substrates [10], [27]–[29], which may include tryptophan. However, OCT1 and OCT2 can be distinguished based on differential affinities for selected substrates and different tissue distributions [10], [30], [31]. OCT1 is expressed primarily in the sinusoidal membrane of hepatocytes, while OCT2 is expressed mainly in the basolateral membrane of kidney proximal tubules. Thus, OCT1 and OCT2 are thought to play critical roles in the biliary and renal excretion of organic cationic substances in the liver and kidney, respectively [32]. In the present study, tryptophan was a stronger inhibiter of OCT2 transport activity than OCT1 transport activity, and OCT2-mediated tryptophan uptake was greater than that mediated by OCT1. Together, the data suggest that compared with OCT1, OCT2 has higher binding affinity and transport activity for tryptophan.

Finally, the absolute quantification of tryptophan by LC-MS/MS showed the highest tryptophan levels in urine samples from *SLC22A2* reference carriers, followed by *808GT* heterozygotes and then *808TT* homozygotes.

Collectively, the findings in the present study indicate that variations of the *SLC22A2* genotype may modulate the uptake of tryptophan. The decreased transport function associated with the *SLC22A2* variants results in decreased renal clearance of tryptophan, leading to decreased renal concentrations of tryptophan, as previously observed for metformin. Additionally, *in vitro* functional studies of the inhibition potential and uptake rate of tryptophan strongly suggest that it is a specific endogenous substrate of OCT2 and consequently a potential biomarker of genotype-dependent OCT2 transport activity. Furthermore, the present study demonstrates that non-targeted metabolite profiling can serve as a robust pre-screening tool for potential transporter substrates, thus reducing the time and effort required for *in vitro* and *in vivo* functional studies.

## Materials and Methods

### 1. Chemicals

[5-^3^H]L-Tryptophan (61 mCi/mmol), *N*-[methyl-^3^H]-4-phenylpyridinium acetate (MPP^+^, 81 Ci/mmol), [^3^H]para-aminohippurate (PAH, 4.6 Ci/mmol), [^3^H]estrone-3-sulfate (ES, 57 Ci/mmol), and [^3^H]taurocholate (TCA, 1.7 Ci/mmol) were purchased from Perkin Elmer Life Science (Boston, MA). Unlabeled tryptophan, L-tryptophan-d_5_, MPP^+^, PAH, ES, and TCA were purchased from Sigma-Aldrich Chemical Co. (St. Louis, MO). Fatty acid methyl esters (retention time index markers: C8, C9, C10, C12, C14, C16, C18, C20, C22, C24, C26, C28, and C30) and 98% pure methoxyamine hydrochloride (CAS No. 593-56-6) were from Sigma-Aldrich. *N*-Methyl-*N*-trimethylsilyl-trifluoracetamide (MSFTA) with 1% trimethylchlorosilan (TMCS) was obtained from Pierce (Rockford, IL). High-performance liquid chromatography-grade acetonitrile and water were purchased from JT Baker (Phillipsburg, NJ). All other chemicals and solvents were of the highest analytical grade available.

### 2. GC-time of flight (TOF) MS analysis for metabolites

Urine samples for metabolomic analysis were obtained from our previous study, which was performed to elucidate the influence of genetic variants of *SLC22A2* on the disposition of metformin [17]. The study protocol was approved by the Institutional Review Board of Inje University Busan Paik Hospital, Busan, Korea. All the subjects provided written informed consent before participating in the study. The genotypes of the subjects were *SLC22A2* reference (*n* = 9), *808GT* heterozygous (*n* = 6), and *808TT* homozygous (*n* = 6). Urine samples were collected for 12 h and stored at −80°C prior to extraction. The endogenous primary metabolites in urine were profiled by GC-TOF as previously described, with some modifications [33]. Briefly, urine aliquots (15 µL) were extracted using 1 mL of acetonitrile∶isopropanol∶water (3∶3∶2; v/v/v) at −20°C, and centrifuged. The supernatants were collected, and the solvent was evaporated to complete dryness. A clean-up step with 500 µL of acetonitrile/water (1∶1; v/v) was applied to remove membrane lipids and triglycerides, and the sample was dried again. A mixture of internal retention index (RI) markers was prepared using fatty acid methyl esters of C8, C9, C10, C12, C14, C16, C18, C20, C22, C24, C26, C28, and C30 linear chain lengths dissolved in chloroform at a concentration of 0.8 mg/ml (C8–C16) or 0.4 mg/mL (C18–C30). A total of 1 µL of RI mixture was added to the dried extracts. Next, 10 µL of a solution of 40 mg/mL of 98% pure methoxyamine hydrochloride in pyridine (silylation grade; Pierce) were added and shaken at 30°C for 90 min, to protect aldehyde and ketone groups. Then, 90 µL of MSTFA with 1% TMCS were added and shaken at 37°C for 30 min, for trimethylsilylation of acidic protons. A Gerstel automatic liner exchange system with a multipurpose sample MPS2 dual rail was used to inject 0.5 µL of the sample into a Gerstel CIS cold injection system (Gerstel, Muehlheim, Germany). The injector was operated in the splitless mode, opening the split vent after 25 s and then ramping up to 250°C. Analytes were separated using an Agilent 6890 gas chromatograph (Santa Clara, CA) equipped with a 30-m long, 0.25-mm i.d. Rtx5Sil-MS column (0.25 µm of 5% diphenyl film and an additional 10-m integrated guard column; Restek, Bellefonte, PA). Mass spectrometry was performed using a Leco Pegasus IV TOF mass spectrometer (St. Joseph, MI) with a transfer line temperature of 280°C, electron ionization at −70 eV, and an ion source temperature of 250°C. Mass spectra were acquired from *m/z* 85 to 500 at 17 spectra s^−1^ and 1850 V detector voltage. The files were preprocessed directly after data acquisition and stored as ChromaTOF-specific *.peg files, generic *.txt result files, or generic ANDI MS *.cdf files. The files were processed using the metabolomics BinBase database [34]. All database entries in BinBase were matched against the Fiehn mass spectra library of ∼1,200 authentic metabolite spectra, using the retention index and mass spectrum information, or the NIST05 commercial library. Identified metabolites were reported when present in at least 80% of the samples per study design group (as defined in the SetupX database) [35].

### 3. Statistical data analysis

Statistical analyses were performed on all continuous variables, which were normalized by excreted urine volume collected for 12 hr, using Statistica software v. 8.0 (StatSoft, Tulsa, OK). In addition data set was normalized with creatinine level and tested for statistical validation of the result. Univariate statistics for multiple study design classes were performed using Student's *t*-test breakdown and one-way ANOVA, and Scheffé's multi-comparison analysis. Data are displayed as box-whisker plots, giving the arithmetic mean for each category with the standard error as a box and whiskers for 1.96 times the category standard error. Multivariate statistics were performed by unsupervised principal component analysis (PCA) and supervised partial least square (PLS) statistics, which require information regarding the assigned study classes. As classical canonical correlation analysis (CCA) cannot be simultaneously performed with large data sets due to numerical limitations (e.g., the numbers of samples and variables), a regularized version of CCA, which is available in the CCA package [36] for the statistical software R, was used. CCA seeks to recognize correlations between two different sources of data sets. Given a set of metabolites and a set of PK parameters of metformin, the primary aim of CCA is to identify two linear combinations, one for the set of metabolites and one for the set of PK parameters that are maximally co-varianced. For visualization, two-dimensional scatter plots (canonical loadings plots) were used for the PK parameters and metabolites. The axes present the canonical variates that can be determined by scree plots. Here, the data were normalized using the values mean-centered and divided by the standard deviation of each variable. As both PK parameters and metabolites are assumed to be of unit variance, their projections on the plane are located within a circle of radius 1 and centered at the origin. Variables with a strong relation are projected in the same direction from the origin, such that the greater the distance from the origin, the stronger is the relation. A second circle with radius of 0.5 is shown to indicate weaker associations of metabolites and PK parameters. For detailed information on the linearity of each variable set (metabolite-PK parameter), a network was generated using pairwise correlation between metabolites and PK parameters [37]. Edges indicate linear associations with cutoff >0.65, and nodes with a circle or square represent metabolites or PK parameters, respectively.

### 4. Assay of tryptophan concentrations using liquid chromatography-tandem mass spectrometry

Tryptophan concentrations in urine samples were quantified using liquid chromatography-tandem mass spectrometry (LC-MS/MS; API 3000; Applied Biosystems, Foster City, CA). Briefly, aliquots of urine samples (40 µL) were extracted by addition of 120 µl of ice-cold methanol containing 1,000 ng/mL tryptophan-d_5_ as an internal standard and 0.13 N HCl. After centrifugation (14,000 rpm for 10 min at 4°C), aliquots (2 µL) of the supernatant were analyzed by LC-MS/MS. The analytes were separated on a reversed-phase column (Atlantis dC18, 2.1 mm i.d.×150 mm, 3-µm particle size; Waters, Milford, MA) with an isocratic mobile phase consisting of acetonitrile and water (30∶70, v/v) containing 0.1% formic acid. The mobile phase was eluted at 0.2 ml/min, using an Agilent 1100 series pump (Agilent, Wilmington, DE). The turbo ion spray interface was operated in the positive ion mode at 5500 V and 400°C. Operating conditions were optimized by flow injection of a mixture of all analytes and were determined as follows: nebulizing gas flow, 40 psi; curtain gas flow, 10 psi; and collision energy, 20 eV. Quadruples Q1 and Q3 were set on unit resolution. Quantitation was performed by selected reaction monitoring of the protonated precursor ion and the related product ion for tryptophan, using the internal standard method. The mass transitions used for tryptophan and tryptophan-d_5_ were *m*/*z* 205→188 and 210→192, respectively. The analytical data were processed using Analyst software (version 1.4). Calibration curves in urine provided a reliable response from 100 to 200,000 ng/mL. Inter-assay precision values for tryptophan analysis in urine samples were less than 15.0%.

### 5. Uptake of tryptophan and MPP^+^ in oocytes expressing OCT2 reference and variant

The synthesis of cRNA and uptake experiments were performed as described previously [27]. Briefly, capped cRNAs were synthesized *in vitro* using T7 RNA polymerase with linear plasmid DNA containing the reference and variants of OCT2 (pcDNA3.1-*SLC22A2*-reference and −808G>T). Defolliculated oocytes were injected with 50 ng of the capped cRNA and incubated at 18°C in Barth's solution (88 mM NaCl, 1 mM KCl, 0.33 mM Ca(NO_3_)_2_, 0.4 mM CaCl_2_, 0.8 mM MgSO_2_, 2.4 mM NaHCO_3_, 10 mM HEPES, pH 7.4) containing 50 µg/mL gentamicin and 2.5 mM pyruvate. After incubation for 2 days, uptake experiments were performed at room temperature in ND96 solution (96 mM NaCl, 2 mM KCl, 1.8 mM CaCl_2_, 1 mM MgCl_2_, 5 mM HEPES, pH 7.4) containing [^3^H]tryptophan (100 nM) or [^3^H]MPP^+^ (18.5 nM). After washing five times, the oocytes were solubilized with 10% SDS, and the radioactivity in the oocytes was analyzed. The uptake of tryptophan and MPP^+^ mediated by the OCT2 reference and variant was calculated by subtracting the uptake rate of water-injected oocytes from that of OCT2-expressing oocytes.

Inhibition of the uptake of 18.5 nM [^3^H]MPP^+^ by various concentrations of TEA (0.01–1 mM), glycine (1–100 mM), taurine (1–100 mM), and tryptophan (0.01–10 mM) was measured in oocytes expressing OCT2. The affinity of tryptophan for OCT2 was assessed by measuring the concentration of tryptophan that inhibited uptake of MPP^+^ by 50% (IC_50_ value). The inhibitory effect of tryptophan on the transport activities of OCT1, OAT1, OATP1B1, and NTCP was determined by measuring the OCT1-mediated MPP^+^ uptake (18.5 nM), OAT1-mediated PAH uptake (400 nM), OATP1B1-mediated ES uptake (18.5 nM), and NTCP-mediated TCA uptake (1.5 µM) in the presence of tryptophan (1 and 10 mM). OCT1, OAT1, OATP1B1, and NTCP were expressed in oocytes as described previously, using linearized plasmids pcDNA3.1-OCT1, -OAT1, -OATP1B1, and -NTCP as templates.

## Supporting Information

Figure S1 **Unsupervised multivariate analysis (PCA) of the** ***SLC22A2*** **reference group and variants.**
(PPTX)

Figure S2 **Visualization of the CCA results for metabolites and metformin PK parameters.** In total, 85 identified and 43 unknown metabolites, which were the most significantly altered (ANOVA, *p*<0.001), were subjected to canonical structure correlation with eight PK parameters. Four compounds (234622, 267701, 267652, and 224529) showed linear associations with PK parameters (canonical correlation, >0.70).
(PPTX)

Figure S3 **Uptake of 100 nM tryptophan by oocytes overexpressing (A) OCT1, (B) OAT1, (C) OATP1B1, and (D) NTCP.** Each bar represents the mean ± SE of eight independent experiments. * *P*<0.05, compared with water-injected oocytes (mock) using Student's *t*-test.
(PPTX)

Spreadsheet S1 **Statistical result-normalized data, Student's** ***t*** **-test, ANOVA, rCCA.**
(XLS)

Text S1 **Statistical validation with creatinine normalization method.**
(DOCX)

## References

[1] Fiehn O (2002). Metabolomics–the link between genotypes and phenotypes.. Plant Molecular Biology.

[2] Assfalg M, Bertini I, Colangiuli D, Luchinat C, Schäfer H (2008). Evidence of different metabolic phenotypes in humans.. Proceedings of the National Academy of Sciences.

[3] Lindon JC, Holmes E, Nicholson JK (2007). Metabonomics in pharmaceutical R & D. Febs Journal.

[4] Griffin JL (2006). The Cinderella story of metabolic profiling: does metabolomics get to go to the functional genomics ball?. Philosophical Transactions of the Royal Society B: Biological Sciences.

[5] Nicholson JK, Connelly J, Lindon JC, Holmes E (2002). Metabonomics: a platform for studying drug toxicity and gene function.. Nature Reviews Drug Discovery.

[6] Kidd D, Liu Y, Cravatt BF (2001). Profiling serine hydrolase activities in complex proteomes.. Biochemistry.

[7] Saghatelian A, Trauger SA, Elizabeth J, Hawkins EG, Siuzdak G (2004). Assignment of endogenous substrates to enzymes by global metabolite profiling.. Biochemistry.

[8] Gieger C, Geistlinger L, Altmaier E, Hrabé de Angelis M, Kronenberg F (2008). Genetics meets metabolomics: a genome-wide association study of metabolite profiles in human serum.. PLoS Genet.

[9] Kimura N, Masuda S, Tanihara Y, Ueo H, Okuda M (2005). Metformin is a superior substrate for renal organic cation transporter OCT2 rather than hepatic OCT1.. Drug Metabolism and Pharmacokinetics.

[10] Koepsell H, Lips K, Volk C (2007). Polyspecific organic cation transporters: structure, function, physiological roles, and biopharmaceutical implications.. Pharm Res.

[11] Okuda M, Saito H, Urakami Y, Takano M, Inui K (1996). cDNA cloning and functional expression of a novel rat kidney organic cation transporter, OCT2.. Biochem Biophys Res Commun.

[12] Fujita T, Urban TJ, Leabman MK, Fujita K, Giacomini KM (2006). Transport of drugs in the kidney by the human organic cation transporter, OCT2 and its genetic variants.. J Pharm Sci.

[13] Kang HJ, Song IS, Shin HJ, Kim WY, Lee CH (2007). Identification and functional characterization of genetic variants of human organic cation transporters in a Korean population.. Drug Metab Dispos.

[14] Leabman MK, Huang CC, Kawamoto M, Johns SJ, Stryke D (2002). Polymorphisms in a human kidney xenobiotic transporter, OCT2, exhibit altered function.. Pharmacogenetics.

[15] Song IS, Shin HJ, Shin JG (2008). Genetic variants of organic cation transporter 2 (OCT2) significantly reduce metformin uptake in oocytes.. Xenobiotica.

[16] Chen Y, Li S, Brown C, Cheatham S, Castro RA (2009). Effect of Genetic Variation in the Organic Cation Transporter 2, OCT2, on the Renal Elimination of Metformin.. Pharmacogenetics and genomics.

[17] Song IS, Shin HJ, Shim EJ, Jung IS, Kim WY (2008). Genetic variants of the organic cation transporter 2 influence the disposition of metformin.. Clin Pharmacol Ther.

[18] Meyer RC, Steinfath M, Lisec J, Becher M, Witucka-Wall H (2007). The metabolic signature related to high plant growth rate in Arabidopsis thaliana.. Proceedings of the National Academy of Sciences.

[19] Jozefczuk S, Klie S, Catchpole G, Szymanski J, Cuadros-Inostroza A (2010). Metabolomic and transcriptomic stress response of Escherichia coli.. Molecular Systems Biology.

[20] Saude EJ, Adamko D, Rowe BH, Marrie T, Sykes BD (2007). Variation of metabolites in normal human urine.. Metabolomics.

[21] Gu H, Pan Z, Xi B, Hainline BE, Shanaiah N (2009). 1H NMR metabolomics study of age profiling in children.. NMR in Biomedicine.

[22] Slupsky CM, Rankin KN, Wagner J, Fu H, Chang D (2007). Investigations of the effects of gender, diurnal variation, and age in human urinary metabolomic profiles.. Analytical chemistry.

[23] Paterson N (1967). Relative constancy of 24-hour urine volume and 24-hour creatinine output.. Clinica chimica acta.

[24] Zamora-Ros R, Rabassa M, Cherubini A, Urpi-Sarda M, Llorach R (2011). Comparison of 24-h volume and creatinine-corrected total urinary polyphenol as a biomarker of total dietary polyphenols in the InCHIANTI Study..

[25] Warrack BM, Hnatyshyn S, Ott KH, Reily MD, Sanders M (2009). Normalization strategies for metabonomic analysis of urine samples.. Journal of Chromatography B.

[26] Nakakariya M, Shima Y, Shirasaka Y, Mitsuoka K, Nakanishi T (2009). Organic anion transporter OAT1 is involved in renal handling of citrulline.. American Journal of Physiology-Renal Physiology.

[27] Choi MK, Song IS (2008). Organic cation transporters and their pharmacokinetic and pharmacodynamic consequences.. Drug Metab Pharmacokinet.

[28] Zhang X, Evans KK, Wright SH (2002). Molecular cloning of rabbit organic cation transporter rbOCT2 and functional comparisons with rbOCT1.. Am J Physiol Renal Physiol.

[29] Zhang L, Dresser MJ, Gray AT, Yost SC, Terashita S (1997). Cloning and functional expression of a human liver organic cation transporter.. Mol Pharmacol.

[30] Arndt P, Volk C, Gorboulev V, Budiman T, Popp C (2001). Interaction of cations, anions, and weak base quinine with rat renal cation transporter rOCT2 compared with rOCT1.. Am J Physiol Renal Physiol.

[31] Kaewmokul S, Chatsudthipong V, Evans KK, Dantzler WH, Wright SH (2003). Functional mapping of rbOCT1 and rbOCT2 activity in the S2 segment of rabbit proximal tubule.. Am J Physiol Renal Physiol.

[32] Jonker JW, Schinkel AH (2004). Pharmacological and physiological functions of the polyspecific organic cation transporters: OCT1, 2, and 3 (SLC22A1-3).. J Pharmacol Exp Ther.

[33] Kind T, Tolstikov V, Fiehn O, Weiss RH (2007). A comprehensive urinary metabolomic approach for identifying kidney cancer.. Analytical Biochemistry.

[34] Fiehn O, Wohlgemuth G, Scholz M (2005). Setup and annotation of metabolomic experiments by integrating biological and mass spectrometric metadata.. Data Integration in the Life Sciences.

[35] Scholz M, Fiehn O (2007). SetupX-a public study design database for metabolomic projects..

[36] González I, Déjean S, Martin PGP, Baccini A (2008). CCA: An R package to extend canonical correlation analysis.. Journal of Statistical Software.

[37] Lê Cao KA, González I, Déjean S (2009). integrOmics: an R package to unravel relationships between two omics datasets.. Bioinformatics.

