# Supplementary figures and images for: Pharmacogenetics Meets Metabolomics: Discovery of Tryptophan as a New Endogenous OCT2 Substrate Related to Metformin Disposition
Source: PLoS One. 2012 May 8;7(5):e36637. doi: 10.1371/journal.pone.0036637 (PMC3348126; doi:10.1371/journal.pone.0036637)

## Slide 1
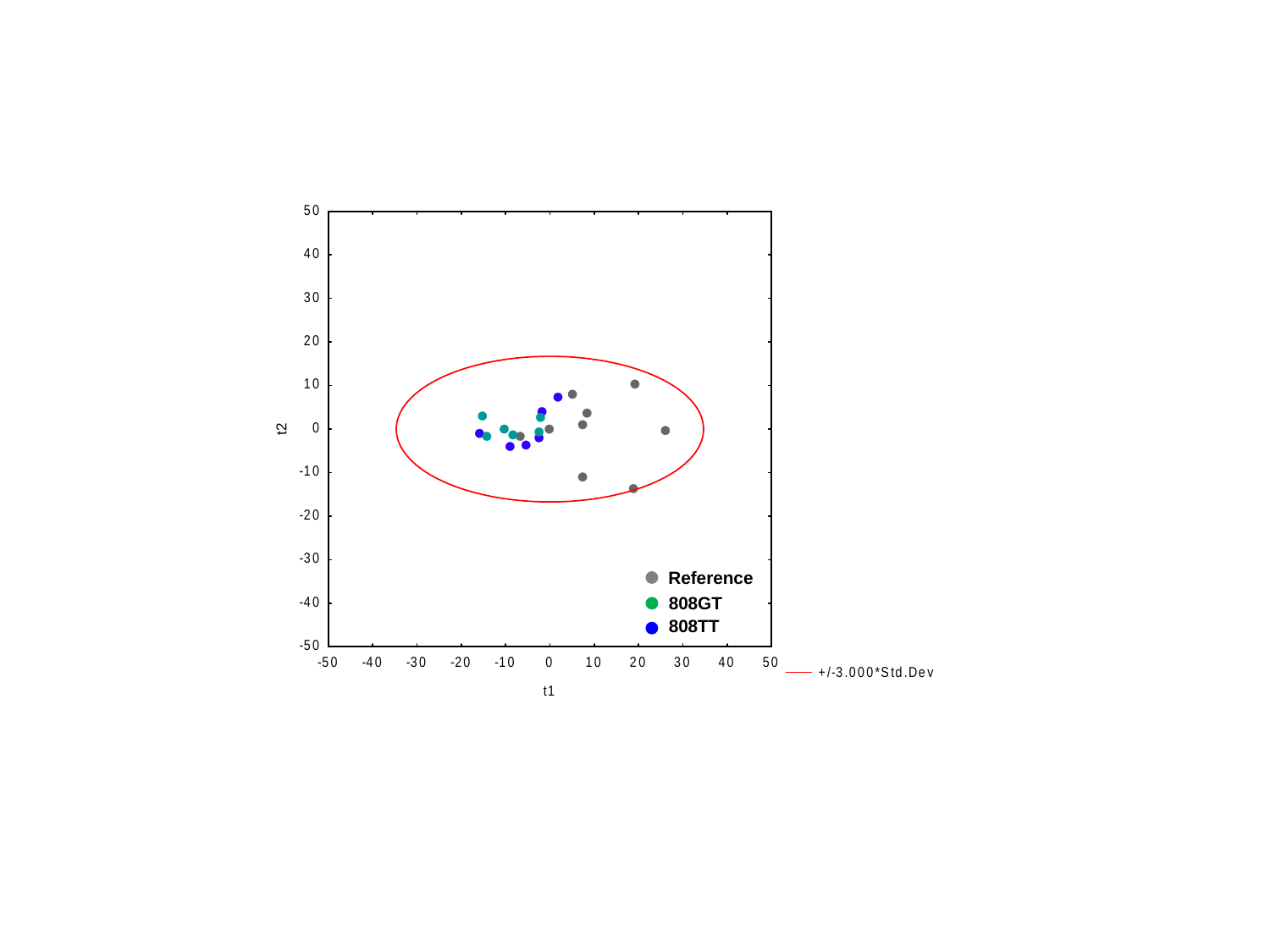

Reference
808GT
808TT

Supplement: Figure S1 — Unsupervised multivariate analysis (PCA) of the SLC22A2 reference group and variants. (PPTX) [file pone.0036637.s001.pptx]

## Slide 1
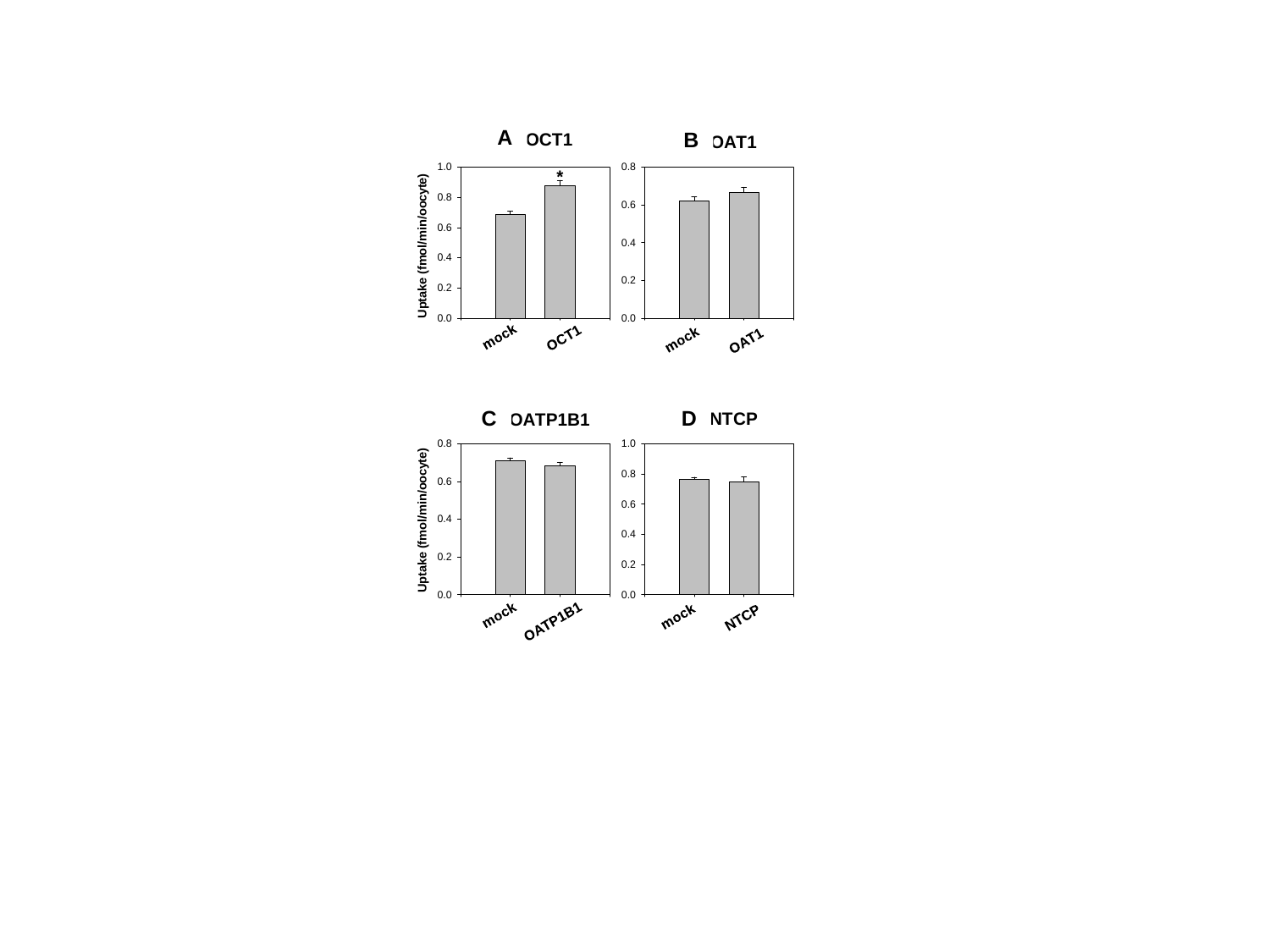

A
B
C
D

Supplement: Figure S3 — Uptake of 100 nM tryptophan by oocytes overexpressing (A) OCT1, (B) OAT1, (C) OATP1B1, and (D) NTCP. Each bar represents the mean ± SE of eight independent experiments. * P<0.05, compared with water-injected oocytes (mock) using Student's t-test. (PPTX) [file pone.0036637.s003.pptx]
