# Supplementary material for: Pharmacogenetics Meets Metabolomics: Discovery of Tryptophan as a New Endogenous OCT2 Substrate Related to Metformin Disposition
Source: PLoS One. 2012 May 8;7(5):e36637. doi: 10.1371/journal.pone.0036637 (PMC3348126; doi:10.1371/journal.pone.0036637)
Supplement: Figure S2 — Visualization of the CCA results for metabolites and metformin PK parameters. In total, 85 identified and 43 unknown metabolites, which were the most significantly altered (ANOVA, p<0.001), were subjected to canonical structure correlation with eight PK parameters. Four compounds (234622, 267701, 267652, and 224529) showed linear associations with PK parameters (canonical correlation, >0.70). (PPTX) [file pone.0036637.s002.pptx]

## Slide 1
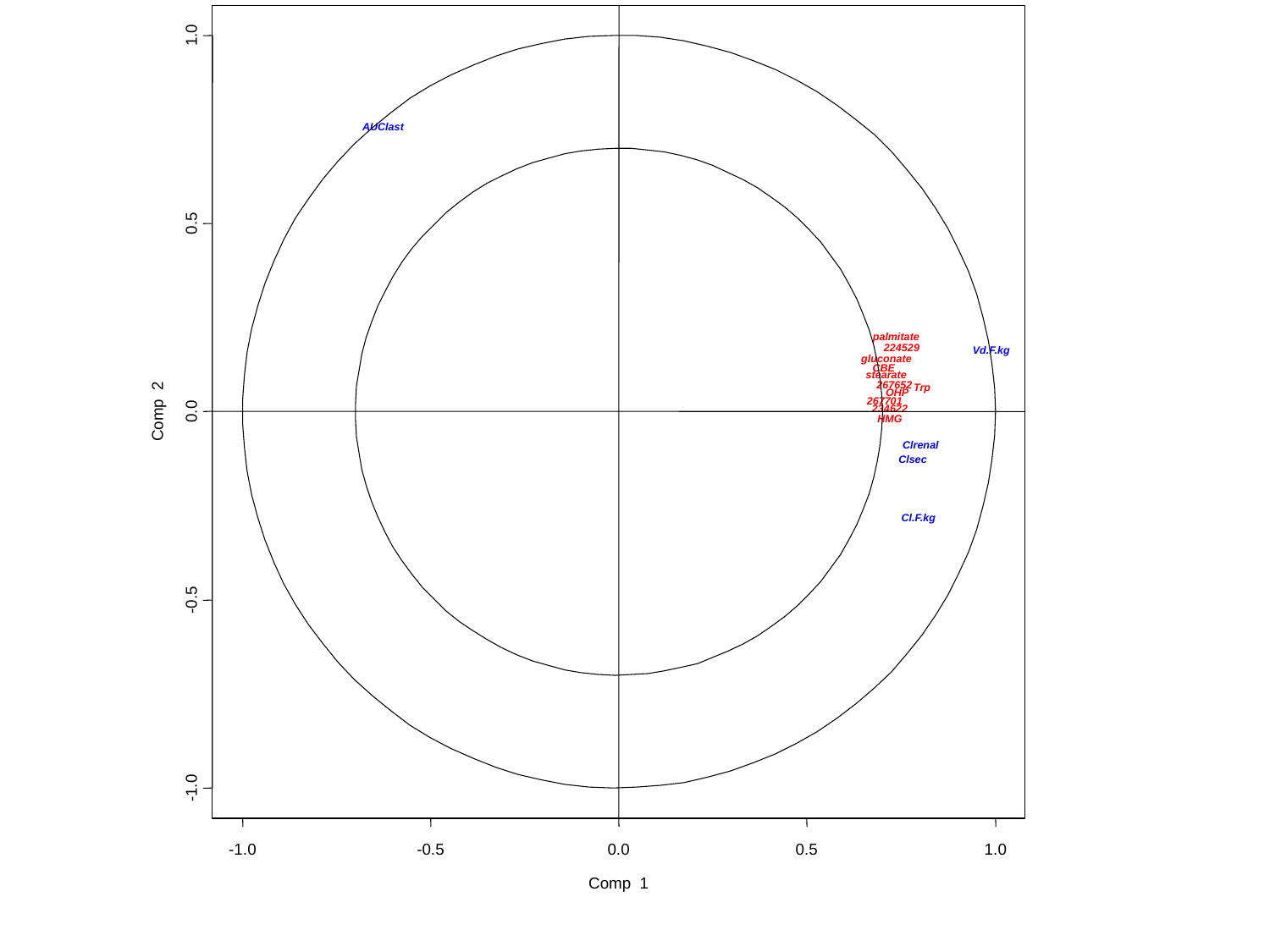

1.0
AUClast
0.5
palmitate
224529
Vd.F.kg
gluconate
CBE
stearate
267652
Trp
OHP
267701
Comp 2
0.0
234622
HMG
Clrenal
Clsec
Cl.F.kg
-0.5
-1.0
-1.0
-0.5
0.0
0.5
1.0
Comp 1
