# Supplementary material for: Pharmacogenetics Meets Metabolomics: Discovery of Tryptophan as a New Endogenous OCT2 Substrate Related to Metformin Disposition
Source: PLoS One. 2012 May 8;7(5):e36637. doi: 10.1371/journal.pone.0036637 (PMC3348126; doi:10.1371/journal.pone.0036637)
Supplement: Text S1 — Statistical validation with creatinine normalization method. (DOCX) [file pone.0036637.s005.docx]

***Validation of normalization method*** we tested the significance of the metabolite profile result by applying other type of data normalization. Along with excreted urine volume, which we currently used for the data analysis in this paper, creatinine normalization has been another frequently used method for diverse quantitation analysis of urine sample. Thus we normalized the data set with creatinine content and compared the result with the one normalized by excreted urine volume. First we performed the linear regression between creatinine and excreted urine volume in order to see if there is coordination of two normalization factors. The resulting scatter plot showed high probability that two normalization factors could be compatible (Figure 1). Next, we performed reproducibility test of biological replicate that is one of the most important factors and that may require the most adequate normalization method. From the result, we found the normalization with urine volume is superior to the one with creatinine content (Table 1). The percentage of coefficient of variation (%CV) laid on around 60 % in the creatinine-normalized dataset while the normalization method with urine volume presented 50 % of CV. rCCA analysis of creatinine-normalized data revealed similar outcome with volume-normalized dataset in which the highest level of association with the PK parameters was found in tryptophan (Figure 3) but not observed in uridine or taurine. Considering altogether, we decided to add taurine which also showed the greatest level of alteration in both normalization methods as did uridine for the following function study. In particular, taurine can be another strong candidate next to tryptophan, which can be applied to practical application as biomarker since it is highly abundant compound in urine so there is great biological variation.

Text S1-Table 1. The comparison of coefficient of variation

|  | **After Normalization based on excreted urine amount** | **After Normalization based on creatinine amount** |
| --- | --- | --- |
| Reference | 53% | 60% |
| Homozygote | 47% | 64% |
| Heterozygote | 51% | 61% |


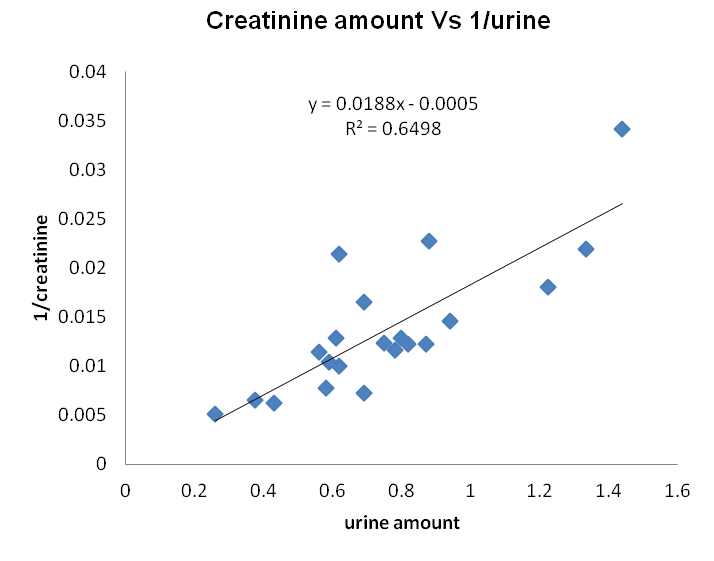


Text S1-Figure 1. Scatter plot


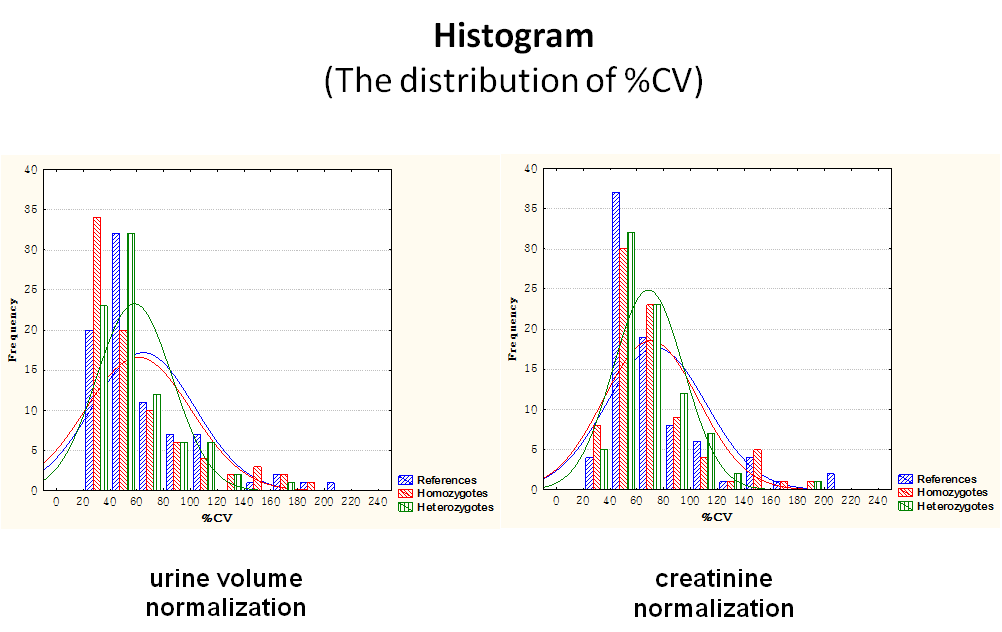


Text S1-Figure 2. The comparison of distribution of %CV of all identified metabolites


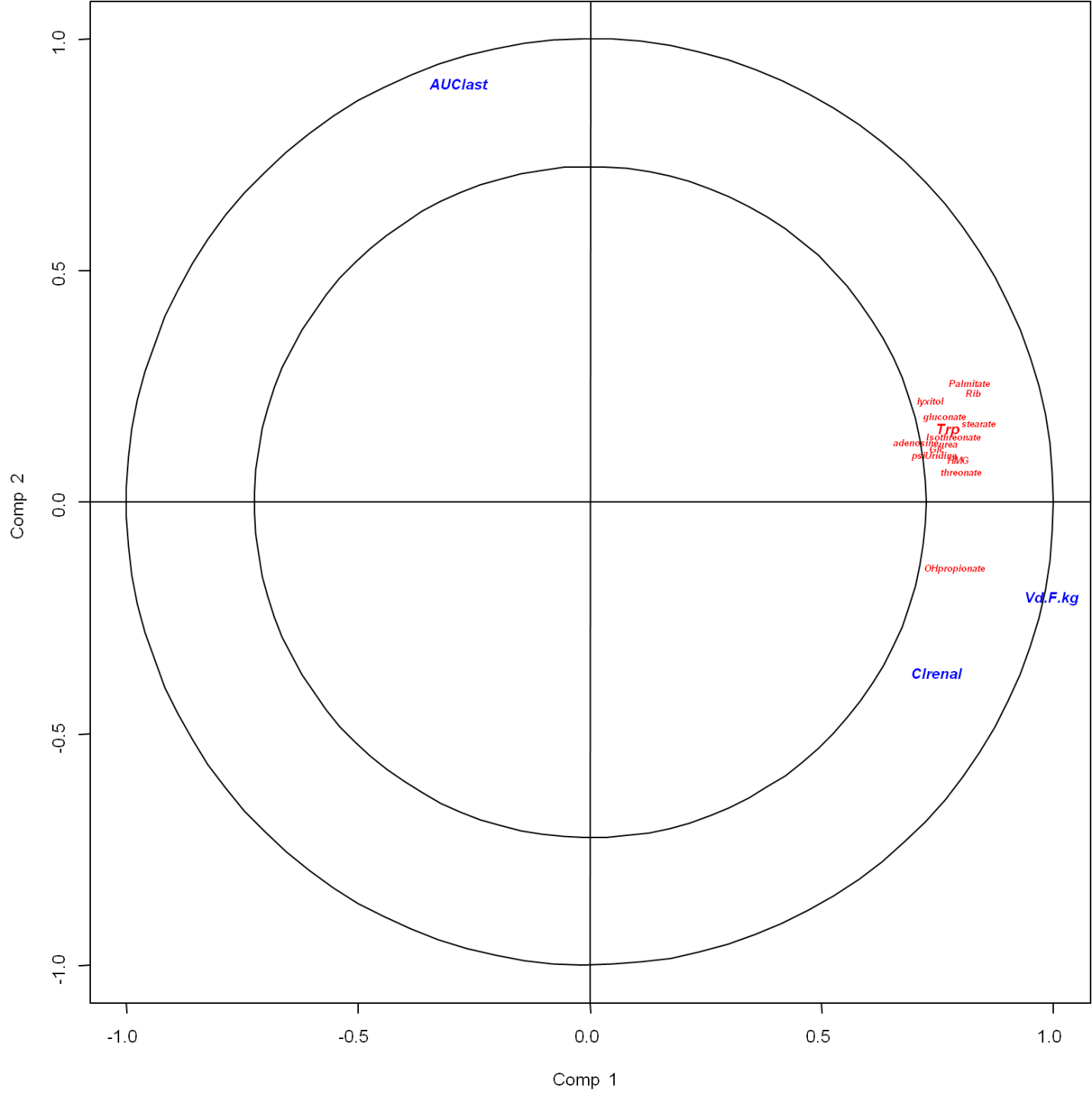


Text S1-Figure 3. rCCA analysis of the dataset normalized by creatinine content. Trp, tryptophan; Rib, ribose; Glc, glucose; HMG, 3-hydroxy-3-methylglutaric acid; OHpropionate, 3-hydroxypropionic acid; psiUridine, pseudo-uridine.
